# Selective parathyroid autotransplantation prevent permanent hypoparathyroidism after total thyroidectomy with central neck dissection

**DOI:** 10.3389/fsurg.2025.1565581

**Published:** 2025-04-24

**Authors:** Haili Sun, Li Gao, Guizhou Xiao, Lei Xie, Yiyu Zhuang, Jianbiao Wang

**Affiliations:** ^1^ Nursing Department, The Affiliated Sir Run Run Shaw Hospital, Zhejiang University School of Medicine, Hangzhou, Zhejiang, China; ^2^ Department of Head and Neck Surgery, The Affiliated Sir Run Run Shaw Hospital, Zhejiang University School of Medicine, Hangzhou, Zhejiang, China

**Keywords:** parathyroid gland, autotransplantation, *in situ* preservation, transient hypoparathyroidism, permanent hypoparathyroidism

## Abstract

**Background:**

The impact of parathyroid gland autotransplantation on permanent hypoparathyroidism remains incompletely understood. This study aimed to ascertain how selective autotransplantation of parathyroid glands affects the occurrence of permanent hypoparathyroidism after total thyroidectomy with central neck dissection (CND).

**Method:**

A retrospective cohort study encompassed consecutive patients with papillary thyroid carcinoma who underwent primary total thyroidectomy plus CND from January 2008 to December 2010 and January 2012 to December 2019. Patients were categorized into two groups (0 and ≥1 parathyroid glands autotransplanted, respectively).

**Result:**

The autotransplantation group comprised 501 patients, while the non-autotransplantation group comprised 652 patients. The autotransplantation group showed significantly lower permanent hypoparathyroidism than the non-autotransplantation group [1.2% (6 of 501) vs. 4.4% (29 of 652), *P* = 0.001]. Out of the total 1,153 patients, 652 (56.5%) had no autotransplanted glands, and 358 (31.0%), 136 (11.8%), and 7 (0.6%) had 1, 2, and 3 glands autotransplanted, respectively. As the number of autotransplanted glands increased (from 0 to 3), the prevalence of permanent hypoparathyroidism was 4.4% (29 of 652), 1.4% (5 of 358), 0.7% (1 of 136), and 0.0% (0 of 7), respectively (*P* = 0.016). Multivariate logistic analysis revealed that parathyroid autotransplantation independently prevented postoperative permanent hypoparathyroidism.

**Conclusion:**

Selective parathyroid autotransplantation is associated with a lower risk of permanent postoperative hypoparathyroidism. Autotransplantation is recommended for parathyroid glands that are devascularized or challenging to preserve in their original location.

## 1 Introduction

Permanent hypoparathyroidism is a troubling long-term complication following total thyroid removal. The reported occurrence ranges from 4% to 11% (1–8). Individuals enduring permanent hypoparathyroidism not only face the inconvenience of daily calcium/vitamin D supplementation but also encounter an elevated risk of renal insufficiency, any malignancy (7), and mortality (8).

Hypoparathyroidism arises from intraoperative harm to the parathyroid glands, such as mechanical or thermal damage, gland devascularization, or unintended removal of parathyroid tissue (9, 10). The general recommendation for averting postoperative parathyroid failure involves a meticulous surgical approach to safeguard the parathyroid glands along with their blood supply *in situ*. However, this proves challenging in practice, even for experienced surgeons with substantial caseloads (11). Due to their anatomical location, parathyroid glands may still be devascularized or occasionally identified in surgical specimens. Autotransplantation of devascularized or inadvertently excised parathyroid glands is commonly employed to prevent enduring postoperative hypoparathyroidism (12, 13). Some authors advocate routine autotransplantation of at least one parathyroid gland or all discernible parathyroid glands to minimize the occurrence of permanent hypoparathyroidism (14–17). Nevertheless, two studies have indicated that parathyroid autotransplantation escalates the risk of enduring hypoparathyroidism (11, 18). A recent meta-analysis has demonstrated that parathyroid autotransplantation does not diminish the occurrence of permanent hypoparathyroidism and that an increased number of autotransplanted parathyroid glands does not correlate with a lower incidence of postoperative enduring hypoparathyroidism (19).

Thus, the impact of parathyroid autotransplantation on postoperative permanent hypoparathyroidism remains unclear. The aim of the current study was to evaluate whether selective parathyroid autotransplantation influences the onset of permanent hypoparathyroidism in a sizable cohort of cases.

## 2 Materials and methods

### 2.1 Patients

A retrospective analysis was conducted on patients diagnosed with papillary thyroid carcinoma (PTC) who underwent total thyroidectomy with ipsilateral or bilateral central neck dissection (CND), including lateral neck dissection, at the Department of Head and Neck Surgery, Affiliated Sir Run Run Shaw Hospital, Zhejiang University School of Medicine, from January 2008 to December 2010, and January 2012 to December 2019. Exclusion criteria comprised prior thyroidectomy, non-PTC cases, procedures less than total thyroidectomy, concurrent parathyroid gland disease, and absence of postoperative parathyroid hormone data.

The study protocol received approval from the Ethics Committee of Affiliated Sir Run Run Shaw Hospital, Zhejiang University School of Medicine.

### 2.2 Surgical decision-making

Following the American Thyroid Association (ATA) guidelines (2009) (20), total thyroidectomy was conducted for patients meeting one of the criteria from January 2008 to December 2015: bilateral nodularity, extra-thyroidal extension, tumor diameter >1.0 cm, multifocal lesions in the affected lobe, and regional or distant metastases. Post January 2016, total thyroidectomy indications were adjusted based on ATA guidelines 2015 (21): thyroid tumors >4 cm, gross extra-thyroidal extension, or clinically apparent metastasis to nodes (clinical N1) or distant sites (clinical M1).

In adherence to the China Thyroid Association expert consensus (22), ipsilateral CND was routinely performed on the affected side in PTC patients, irrespective of clinical metastasis in central neck lymph nodes. Bilateral CND was carried out in cases with tumor(s) in the isthmus and/or both thyroid lobes or in individuals with clinically apparent neck lymph node metastasis.

### 2.3 Surgical procedures

All procedures were conventional open surgeries conducted by three senior surgeons. Thyroidectomy strictly followed the capsular dissection technique recommended by Thompson et al. (23). Parathyroid glands were consistently identified and preserved under direct vision, except for any not found. Vascular supply assessment of parathyroid glands by surgeons relied on gland color and plumpness from January 2008 to December 2010. Post January 2012, blood supply confirmation employed the fine-needle pricking (FNP) test (24). Resected thyroid lobe specimens and fibrofatty tissue from the central compartment were scrutinized for inadvertently removed parathyroid tissue. Any devascularized or unintentionally removed parathyroid gland was cut into small granules and mixed with isotonic sodium chloride solution (0.3–0.5 ml). The suspension was aspirated into a 1-ml syringe and injected into the sternocleidomastoid muscle through the pinhead of a 20 ml syringe (25). Per ATA guidelines, bilateral CND involved removal of prelaryngeal, pretracheal, and both right and left paratracheal nodal basins; unilateral CND entailed removal of prelaryngeal, pretracheal, and the single paratracheal nodal basin (26).

### 2.4 Clinical parameters

Collected parameters comprised sex, age, maximum tumor size, extra-thyroidal extension, multifocality, concurrent thyroiditis, type of central neck dissection (CND), number of harvested and metastatic lymph nodes in the central compartment, T and N classification, the American Joint Committee on Cancer (AJCC) stage (8th edition) (27), number of parathyroid gland autotransplantations, and number of inadvertently excised parathyroid glands. Verification of inadvertently excised parathyroid glands was conducted through the examination of paraffin-embedded specimens from thyroid lobes and fibrofatty tissue in the central compartment.

### 2.5 Laboratory assays

Serum calcium and intact parathyroid hormone (iPTH) levels were assessed in patients before surgery and every morning (06:00 a.m.) postoperative until discharge. Most patients were discharged on postoperative day 4, contingent on drainage fluid volume. Serum iPTH levels were measured using a Roche Cobas E601 instrument (Hitachi High-Technologies, Tokyo, Japan) (normal range 15–65 ng/L). Serum calcium levels were measured on an Abbott Aeroset Automated Instrument Analyzer (Toshiba Medical Systems, Tochigi-ken, Japan) (normal range 2.11–2.52 mmol/L). Ionized calcium amounts were not separately determined in this study.

### 2.6 Definition of hypoparathyroidism

Transient hypoparathyroidism was defined as subnormal serum iPTH level, serum calcium level below 2 mmol/L (8 mg/dl), or the need for calcium supplementation to alleviate clinical symptoms of hypocalcemia (e.g., numbness, paraesthesia, or carpopedal spasm) during the hospital stay (11, 28). Permanent hypoparathyroidism was defined as persistently subnormal serum iPTH level or hypocalcemia six months post-surgery, necessitating calcium and vitamin D supplements (4). Symptomatic hypocalcemia was treated with twice-daily oral calcium supplements containing 600 mg calcium carbonate plus 125 units of vitamin D3. Calcium gluconate injection was prescribed for persistent symptomatic hypocalcemia after oral calcium treatment. All patients were followed up for more than 6 months postoperatively to confirm the presence of permanent hypoparathyroidism.

### 2.7 Statistical analysis

Categorical data were summarized using frequencies and percentages, while continuous data were presented as mean (s.d.) for normally distributed variables or median (range) for non-normally distributed variables. Normal distribution was assessed with the Kolmogorov–Smirnov test. The *χ*^2^ test was employed for categorical variable comparisons. Continuous variable comparisons utilized Student's *t*-test (for normally distributed two samples), Mann–Whitney *U*-test (for non-normally distributed two samples), and Kruskal–Wallis test (for four samples). Variables demonstrating statistical significance in univariate analysis were subjected to multivariate analysis using binary logistic regression to identify risk factors for transient or permanent hypoparathyroidism. Multivariate analysis results were expressed as odds ratio (OR) and 95% confidence interval (CI). All *P* values were two-sided, with a significance level set at <0.05. The analyses were executed using SPSS® version 16.0 (IBM, Armonk, New York, USA).

## 3 Results

### 3.1 Patient characteristics

A total of 1,153 patients met the study criteria, with 501 patients in the autotransplantation group (at least one parathyroid gland autotransplanted) and the remaining 652 patients in the non-autotransplantation group (no parathyroid gland autotransplanted). All patients underwent total thyroidectomy with CND for PTC, with 712 (61.8%) undergoing unilateral CND and 441 (38.2%) undergoing bilateral CND. Simultaneous lateral neck dissections were performed in 219 patients.

Patient characteristics are presented in Table 1. Of the total, 904 were female, and 249 were male, with a mean age of 44.9 years. No differences were observed in the incidence of central neck lymph node metastases, with 262 of 501 autotransplantation patients (52.3%) and 334 of 652 non-autotransplantation patients (51.2%) having such metastases. Age, sex, tumor size, presence of Hashimoto's thyroiditis, N classification, and AJCC stage showed no significant differences between the two groups.

**Table 1:** Baseline characteristics and surgical results for patients undergoing total thyroidectomy and central neck dissection.

Parameter	Autotransplantation Group (*n* = 501)	Non-autotransplantation Group (*n* = 652)	*P* ^‡^
Age (year)*	45.7 (11.9)	44.2 (11.0)	0.054^§^
Sex ratio (F:M)	387: 114	517: 135	0.402
Tumor size on histology (cm)^†^	0.90 (0.15–6.80)	0.90 (0.10–5.00)	0.479^§^
Multifocal lesion	386 (77.0)	299 (45.9)	<0.001
Extra-thyroidal extension	93 (18.6)	57 (8.7)	<0.001
Type of CND			
Unilateral	211 (42.1)	501 (76.8)	<0.001
Bilateral	290 (57.9)	151 (23.2)	
Hashimoto's thyroiditis	92 (18.4)	92 (14.1)	0.051
Overall lymph node yield in CND^†^			
Retrieved	11.0 (0.0–43.0)	9.0 (0.0–37.0)	<0.001^§^
Metastatic	1.0 (0.0–16.0)	1.0 (0.0–21.0)	0.502^§^
Central neck lymph node metastases	262 (52.3)	334 (51.2)	0.719
T classification			
T1–T2	461 (92.0)	621 (95.2)	0.024
T3–T4	40 (8.0)	31 (4.8)	
N classification			
N0	231 (46.1)	298 (45.7)	0.892
N1	270 (53.9)	354 (54.3)	
AJCC stage			
I–II	498 (99.4)	650 (99.7)	0.454
III–IV	3 (0.6)	2 (0.3)	
Parathyroid excised inadvertently	103 (20.6)	211 (32.4)	<0.001
Transient hypoparathyroidism	336 (67.1)	325 (49.8)	<0.001
Permanent hypoparathyroidism	6 (1.2)	29 (4.4)	0.001

Values in parentheses represent percentages unless indicated otherwise: values are *mean (standard deviation) and ^†^median (range). CND, central neck dissection; AJCC, American joint committee on cancer (8th edition). ^‡^Pearson *χ*^2^ test, except ^§^Mann–Whitney *U*-test.

More patients in the autotransplantation group exhibited multifocal lesions, extra-thyroidal extension, bilateral CND, and T3–T4 classification than in the non-autotransplantation group. Although there was no difference in the number of metastatic lymph nodes in CND between the two groups, significantly more lymph nodes were removed in the autotransplantation group than in the non-autotransplantation group. The rate of inadvertently excised parathyroid glands was significantly lower in the autotransplantation group than in the non-autotransplantation group [20.6% (103 of 501) vs. 32.4% (211 of 652), *P* < 0.001].

### 3.2 The prevalence of transient and permanent hypoparathyroidism

In the autotransplantation group, 336 of 501 patients (67.1%) experienced transient hypoparathyroidism, compared to 325 of 652 patients (49.8%) in the non-autotransplantation group (*P* < 0.001). Permanent hypoparathyroidism occurred in 6 patients (1.2%) in the autotransplantation group and 29 patients (4.4%) in the non-autotransplantation group (*P* = 0.001). While parathyroid autotransplantation increased the incidence of transient hypoparathyroidism, the autotransplantation group showed significantly lower permanent hypoparathyroidism.

Among 651 parathyroid glands that underwent autotransplantation, 274 (42.1%) glands were transplanted from inadvertently removed glands, and 377 (57.9%) glands were removed due to devascularization. The number of autotransplanted parathyroid glands was as follows: 0 gland in 56.5% patients (652 of 1,153; group 0), 1 gland in 31.0% patients (358 of 1,153; group 1), 2 glands in 11.8% patients (136 of 1,153; group 2), and 3 glands in 0.6% patients (7 of 1,153; group 3). None of the patients underwent autotransplantation of 4 parathyroid glands (Table 2). The number of parathyroids identified during each surgery among the four groups has no significantly difference (*P* = 0.178) (Table 2). The incidence of transient hypoparathyroidism was 49.8% (325 of 652), 59.5% (213 of 358), 86.0% (117 of 136), and 85.7% (6 of 7) for groups with 0, 1, 2, and 3 parathyroid glands autotransplanted, respectively (*P* < 0.001). The incidence of permanent hypoparathyroidism in these groups was 4.4% (29 of 652), 1.4% (5 of 358), 0.7% (1 of 136), and 0.0% (0 of 7), respectively (*P* = 0.016). The group 0 patients had lowest incidence of transient hypoparathyroidism, but highest prevalence of permanent hypoparathyroidism.

**Table 2:** Comparison of different numbers of parathyroid gland autotransplantation among patients undergoing total thyroidectomy plus CND.

Parameter	Numbers of parathyroid gland autotransplantation				*P* ^‡^
	0 gland (Group 0, *n* = 652)	1 gland (Group 1, *n* = 358)	2 glands (Group 2, *n* = 136)	3 glands (Group 3, *n* = 7)	
Age (year)*	44.2 (11.0)	45.9 (11.9)	45.5 (12.1)	41.9 (9.7)	0.206^§^
Sex ratio (F:M)	517: 135	277: 81	104: 32	6: 1	0.789
Tumor size on histology (cm)^†^	0.90 (0.10–5.00)	0.90 (0.15–6.80)	0.80 (0.30–4.50)	0.80 (0.40–1.50)	0.729^§^
Multifocal lesion	299 (45.9)	259 (72.3)	120 (88.2)	7 (100.0)	<0.001
Extra-thyroidal extension	57 (8.7)	58 (16.2)	33 (24.3)	2 (28.6)	<0.001
Type of CND					<0.001
Unilateral	501 (76.8)	189 (52.8)	22 (16.2)	0 (0.0)	
Bilateral	151 (23.2)	169 (47.2)	114 (83.8)	7 (100.0)	
Hashimoto's thyroiditis	92 (14.1)	64 (17.9)	27 (19.9)	1 (14.3)	0.241
Overall lymph node yield in CND^†^					
Retrieved	9.0 (0.0–37.0)	11.0 (0.0–31.0)	12.0 (1.0–43.0)	10.0 (4.0–28.0)	<0.001^§^
Metastatic	1.0 (0.0–21.0)	1.0 (0.0–14.0)	1.0 (0.0–16.0)	2.0 (0.0–15.0)	0.816^§^
Central neck lymph node metastases	334 (51.2)	189 (52.8)	69 (50.7)	4 (57.1)	0.948
T classification					
T1–T2	621 (95.2)	332 (92.7)	124 (91.2)	5 (71.4)	0.013
T3–T4	31 (4.8)	26 (7.3)	12 (8.8)	2 (28.6)	
N classification					
N0	298 (45.7)	162 (45.3)	67 (49.3)	2 (28.6)	0.674
N1	354 (54.3)	196 (54.7)	69 (50.7)	5 (71.4)	
AJCC stage					
I–II	650 (99.7)	357 (99.7)	134 (98.5)	7 (100.0)	0.277
III–IV	2 (0.3)	1 (0.3)	2 (1.5)	0 (0.0)	
Parathyroid excised inadvertently	211 (32.4)	71 (19.8)	32 (23.5)	0 (0.0)	<0.001
Parathyroid glands identified^†^	4.0 (1.0–4.0)	4.0 (1.0–4.0)	4.0 (2.0–4.0)	4.0 (3.0–4.0)	0.178^§^
Transient hypoparathyroidism	325 (49.8)	213 (59.5)	117 (86.0)	6 (85.7)	<0.001
Permanent hypoparathyroidism	29 (4.4)	5 (1.4)	1 (0.7)	0 (0.0)	0.016

Values in parentheses represent percentages unless indicated otherwise: values are *mean (standard deviation) and ^†^median (range). CND, central neck dissection; AJCC, American joint committee on Cancer (8th edition). ^‡^Pearson *χ*^2^ test, except ^§^Kruskal–Wallis test.

### 3.3 Comparison between autotransplantation and non-autotransplantation groups among patients without inadvertently parathyroidectomy

To eliminate the impact of unintentional parathyroid gland resection on hypoparathyroidism incidence, a subgroup analysis was conducted on patients with no inadvertently excised parathyroid glands (Table 3). In this subgroup, there were 398 and 441 patients in the autotransplantation and non-autotransplantation groups, respectively. The incidence of transient hypoparathyroidism in the autotransplantation group was significantly higher than in the non-autotransplantation group [66.8% (266 of 398) vs. 47.2% (208 of 441), *P* < 0.001], while permanent hypoparathyroidism was significantly lower [0.3% (1 of 398) vs. 3.2% (14 of 441), *P* = 0.001)]. No differences were observed in age, sex, tumor size, presence of Hashimoto's thyroiditis, number of metastatic lymph nodes, T and N classification, and AJCC stage among patients without inadvertent parathyroidectomy in the autotransplantation vs. non-autotransplantation groups (Table 3). However, more patients in the autotransplantation group had multifocal lesions, extra-thyroidal extension, bilateral CND, and a higher number of retrieved lymph nodes compared to the non-autotransplantation group (Table 3).

**Table 3:** Comparison between autotransplantation and Non-autotransplantation groups among patients without inadvertent parathyroidectomy.

Parameter	Autotransplantation Group (*n* = 398)	Non-autotransplantation Group (*n* = 441)	*P* ^‡^
Age (year)*	45.1 (11.8)	43.6 (11.4)	0.051^§^
Sex ratio (F:M)	306: 92	359: 82	0.107
Tumor size on histology (cm)^†^	0.90 (0.15–4.50)	1.00 (0.10–5.00)	0.207^¶^
Multifocal lesion	296 (74.4)	203 (46.0)	<0.001
Extrathyroidal extension	69 (17.3)	39 (8.8)	<0.001
Type of CND			
Unilateral	175 (44.0)	330 (74.8)	<0.001
Bilateral	223 (56.0)	111 (25.2)	
Hashimoto's thyroiditis	76 (19.1)	68 (15.4)	0.159
Overall lymph node yield in CND^†^			
Retrieved	11.0 (0.0–43.0)	10.0 (0.0–37.0)	<0.001^¶^
Metastatic	1.0 (0.0–16.0)	1.0 (0.0–16.0)	0.474^¶^
Central neck lymph node metastases	211 (53.0)	225 (51.0)	0.564
T classification			
T1–T2	371 (93.2)	417 (94.6)	0.417
T3–T4	27 (6.8)	24 (5.4)	
N classification			
N0	180 (45.2)	202 (45.8)	0.866
N1	218 (54.8)	239 (54.2)	
AJCC stage			
I–II	396 (99.5)	439 (99.5)	0.918
III–IV	2 (0.5)	2 (0.5)	
Transient hypoparathyroidism	266 (66.8)	208 (47.2)	<0.001
Permanent hypoparathyroidism	1 (0.3)	14 (3.2)	0.001

Values in parentheses represent percentages unless indicated otherwise: values are *mean (standard deviation) and ^†^median (range). CND, central neck dissection; AJCC, American Joint Committee on Cancer (8th edition). ^‡^Pearson *χ*^2^ test, except ^§^Student's *t* test and ^¶^Mann–Whitney *U*-test.

### 3.4 Risk factors of transient and permanent hypoparathyroidism in patients underwent total thyroidectomy with CND

The patients were categorized into three groups: Normal, Transient and Permanent. Separate univariate and multivariate analyses were conducted to compare “Transient vs. Normal” and “Permanent vs. Transient” groups. Univariate analysis revealed five significant variables associated with transient hypoparathyroidism compared to normal (sex, multifocal lesion, type of CND, metastatic lymph nodes in the central compartment, and parathyroid autotransplantation), while two variables showed significance for permanent vs. transient hypoparathyroidism (inadvertent parathyroid excision and parathyroid autotransplantation) (Table 4).

**Table 4:** Univariate analysis of risk factors for transient and permanent hypoparathyroidism in patients undergoing total thyroidectomy plus CND.

Variables	Normal (*n* *=* 457)	Transient (*n* = 661)	Permanent (*n* = 35)	*P* ^†^	*P* ^‡^
Age (<55/≥55)	357/100	517/144	29/6	0.969	0.515
Sex (male/female)	117/340	125/536	7/28	0.008	0.873
Tumor size on histology (cm)*	0.90 (0.10–6.80)	0.90 (0.10–5.00)	0.90 (0.40–4.70)	0.818^§^	0.513^§^
Multifocal lesion	231	430	24	<0.001	0.670
Extra-thyroidal extension	57	89	4	0.629	0.730
Type of CND (UCND/BCND)	347/110	347/314	18/17	<0.001	0.902
Hashimoto's thyroiditis	68	111	5	0.391	0.698
Harvested lymph nodes in CND (<10/≥10)	218/239	292/369	17/18	0.244	0.610
Harvested lymph nodes in CND (<5/≥5)	86/371	77/584	4/31	0.001	0.968
Metastatic lymph nodes in CND (<5/≥5)	405/52	560/101	31/4	0.062	0.535
Metastatic lymph nodes in CND (0/≥1)	235/222	308/353	14/21	0.112	0.446
T classification (T1–T2/T3–T4)	429/28	620/41	33/2	0.959	0.907
N classification (N0/N1)	223/234	292/369	14/21	0.128	0.628
AJCC stage (I–II/III–IV)	455/2	658/3	35/0	0.968	0.690
Parathyroid excised inadvertently	109	185	20	0.122	<0.001
Parathyroid gland autotransplantation (0/≥1)	298/159	325/336	29/6	<0.001	<0.001

* Median (range). CND, central neck dissection; AJCC, American joint committee on cancer (8th edition). ^†^Normal vs. transient, ^‡^Transient vs. Permanent. ^†, ‡^Pearson *χ*^2^ test, except ^§^Mann–Whitney *U*-test.

Multivariate logistic regression analysis identified female sex, bilateral CND, and parathyroid autotransplantation as independent risk factors for transient hypoparathyroidism (Table 5). Notably, inadvertent parathyroid excision was predictive of progression from transient to permanent hyporathyroidism, whereas parathyroid autotransplantation acted as a protective factor against this progression (Table 6).

**Table 5:** Multivariate logistic regression analysis of risk factors for the development of normal to transient hypoparathyroidism in patients undergoing total thyroidectomy plus CND.

Independent variables	OR	95% CI	*P* value
Sex (female vs. male)	1.487	1.103–2.005	0.009
Multifocal lesion (positive vs. negative)	1.047	0.779–1.406	0.762
Type of CND (BCND vs. UCND)	2.348	1.710–3.224	<0.001
Metastatic lymph nodes in CND (≥5 vs. <5)	1.327	0.914–1.927	0.137
Parathyroid gland autotransplantation (≥1 vs. 0)	1.451	1.108–1.900	0.007

CND, central neck dissection; BCND, bilateral central neck dissection; UCND, unilateral central neck dissection; OR, odds ration; 95% CI, 95% confidence interval.

**Table 6:** Multivariate logistic regression analysis of risk factors for the development of transient to permanent hypoparathyroidism in patients undergoing total thyroidectomy plus CND.

Independent variables	OR	95% CI	*P* value
Parathyroid excised inadvertently (positive vs. negative)	2.810	1.393–5.670	0.004
Parathyroid gland autotransplantation (≥1 vs. 0)	0.234	0.095–0.576	0.002

CND, central neck dissection; OR, odds ration; 95% CI, 95% confidence interval.

## 4 Discussion

Permanent hypoparathyroidism constitutes a significant complication following total thyroidectomy with CND, presenting a challenge for most surgeons. Our study reveals that parathyroid autotransplantation heightens the occurrence of transient hypoparathyroidism but substantially diminishes the risk of permanent hypoparathyroidism. Multivariate analysis underscores parathyroid autotransplantation as a preventive factor against the progression from transient to permanent hyporathyroidism.

Undoubtedly, parathyroid autotransplantation stands as an efficacious method to restore parathyroid gland function after devascularization or identification in surgical specimens (13, 29). Numerous studies demonstrate the long-term survival of the majority of parathyroid grafts (17, 30). Presently, a widespread consensus associates parathyroid autotransplantation during total thyroidectomy with elevated rates of postoperative transient hypoparathyroidism (11, 14, 15, 31–37). Consequently, the primary controversy centers on its potential to prevent permanent hypoparathyroidism.

Some investigations assert that autotransplanting at least one parathyroid gland can reduce or eliminate permanent hypoparathyroidism (14, 15, 30, 33, 38). For instance, Yuxuan Qiu and colleagues demonstrated that the autotransplantation of one or two parathyroid glands independently increased the risk of transient hypoparathyroidism while decreasing the risk of permanent hypoparathyroidism (38). Our study similarly reveals that, although parathyroid autotransplantation increases the incidence of transient hypoparathyroidism, it significantly diminishes the risk of permanent hypoparathyroidism. Multivariate analysis highlights parathyroid autotransplantation as the sole protective factor for permanent hypoparathyroidism.

Contrastingly, other studies argue that parathyroid autotransplantation does not reduce the frequency of permanent hypoparathyroidism (31, 39–43). A meta-analysis concurs, indicating that parathyroid autotransplantation exerts no influence on the risk of permanent hypoparathyroidism (19). Furthermore, two studies suggest that parathyroid autotransplantation heightens the risk of permanent hypoparathyroidism (11, 18). Lorente-Poch L and colleagues report that autotransplantation not only correlates with higher rates of postoperative hypocalcemia but also entails a three-fold increase in permanent hypoparathyroidism rates (11). It's noteworthy that only 117 out of a total of 657 patients underwent CND in the study by Lorente-Poch L, and the authors did not clarify the incidence of inadvertent parathyroidectomy between the autotransplantation and non-autotransplantation groups.

The controversy surrounding the impact of parathyroid autotransplantation on permanent hypoparathyroidism primarily stems from three key issues.

Firstly, the subjective and inconclusive nature of surgeons' assessments of parathyroid gland blood supply contributes to the debate. Relying on observations of gland color and plumpness proves unreliable. Autotransplanted parathyroid glands exhibit more predictable functionality compared to those left *in situ* without blood supply. It is crucial to assess whether a parathyroid gland preserved *in situ* after thyroidectomy is “live” (vascularized) or not. If a devascularized parathyroid gland (appearing intact) is autotransplanted, the likelihood of the patient experiencing permanent hypoparathyroidism is reduced compared to preserving the devascularized gland *in situ*.

Secondly, the lack of uniformity in the technique of parathyroid autotransplantation across different studies adds complexity to the evaluation. Variations in procedural steps, such as whether the gland is immediately autografted (14, 40, 44) or at the end of thyroidectomy (11, 15, 30, 34, 35), the chosen autotransplantation technique (slicing vs. injecting a solution of suspended parathyroid tissue) (45), and the surgeon's criteria for carrying out autotransplantation (routine vs. selective), make it challenging to determine the substantial differences in outcomes.

Thirdly, the differing incidence of inadvertent parathyroidectomy between autotransplantation and non-autotransplantation groups can influence the effect of parathyroid autotransplantation on permanent hypoparathyroidism. Inadvertent parathyroidectomy is a relatively common finding in pathology specimens after thyroid operation. Sitges-Serra et al. reported a 28% prevalence in their specimens after total thyroidectomy with CND (46). Mayer AW et al. reported parathyroid tissue was removed inadvertently in 50 of 378 hemithyroidectomies (13.2%), 73 of 404 near-total or total thyroidectomies (18.1%), 89 of 743 thyroidectomies without CND (12.0%), and 76 of 154 thyroidectomies with CND (49.4%) (47). Other studies of varying extent of thyroid surgery have reported rates between 5.8% and 28% (46–53). The incidence of inadvertent parathyroidectomy, of 27.2% (314 of total 1,153 patients) reported in this study after total thyroidectomy with CND was in keeping with these previously reported series. Certain studies showed inadvertent parathyroidectomy increases the risk of permanent hypoparathyroidism (36, 46, 53). In this study, multivariate analysis identified inadvertent parathyroidectomy as the sole independent risk factor for permanent hypoparathyroidism.

The rate of inadvertently excised parathyroid glands in the non-autotransplantation group was higher than in the autotransplantation group [32.4% (211 of 652) vs. 20.6% (103 of 501), *P* < 0.001] in our study. To eliminate the impact of unintentional parathyroid gland resection on hypoparathyroidism incidence, we compared the prevalence of permanent hypoparathyroidism on patients with no inadvertently excised parathyroid glands. The incidence of permanent hypoparathyroidism in the autotransplantation group was also significantly lower than in the non-autotransplantation group [0.3% (1 of 398) vs. 3.2% (14 of 441), *P* = 0.001)] in these subgroup patients.

FNP test emerges as a simple and reliable tool for evaluating the blood supply of the parathyroid gland (24). Every parathyroid gland preserved *in situ* during thyroidectomy was suggested to undergo FNP testing. Parathyroid glands with no blood supply, as determined by the FNP test, should be promptly autotransplanted into muscle, presenting an effective method to prevent permanent hypoparathyroidism. Current evidence suggests that both indocyanine green (ICG) imaging with SPY camera and parathyroid autofluorescence serve as reliable techniques for evaluating the vascularity and viability of *in situ* preserved parathyroid glands (54–56). Future comparative studies are warranted to evaluate the efficacy of FNP test against ICG imaging and parathyroid autofluorescence in assessing the perfusion status and functional integrity of *in situ* parathyroid glands.

The “thymus-blood vessel-inferior parathyroid gland” layer concept proves to be an effective method for preserving the inferior parathyroid gland (IPTG) *in situ* during CND (57). However, the IPTG and its blood supply are often implicated in dorsal extra-thyroidal invasion of the primary tumor or extra-nodal metastasis of paratracheal lymph nodes. Consequently, autotransplantation of IPTGs and *en bloc* resection of the thyroid and central neck lymph nodes are recommended for these patients. Autotransplanting IPTGs during the initial operation in PTC patients with high-volume central neck lymph node metastasis may prevent permanent hypoparathyroidism after subsequent reoperation of the central neck compartment (58).

The present study has several limitations. Firstly, the data are derived from a retrospective chart review. More patients in the autotransplantation group exhibited multifocal lesions, extra-thyroidal extension, bilateral CND, and T3-T4 classification than in the non-autotransplantation group. These different characteristics between the two groups may cause bias. Secondly, from January 2008 to December 2010, the state of parathyroid gland blood supply was subjectively judged by surgeons based on the gland's color and plumpness, potentially introducing selection bias. Thirdly, the duration of follow up is 6 month postoperatively to identify the presence of permanent hypoparahtyoidism, which is short. Finally, the function of autotransplanted glands cannot be assessed in our study. The recovery of postoperative serum iPTH levels may predominantly depend on the *in situ* preserved parathyroid glands, as no patients underwent autotransplantation of all four parathyroid glands.

## 5 Conclusion

Selective parathyroid autotransplantation appears to increase the incidence of transient hypoparathyroidism after total thyroidectomy with CND, but it serves as a preventive factor for permanent hypoparathyroidism. Autotransplantation is advisable for parathyroid glands that are devascularized or challenging to preserve *in situ*.

## Data Availability

The datasets presented in this study can be found in online repositories. The names of the repository/repositories and accession number(s) can be found below: The data that support the findings of this study are available in the following link. https://doi.org/10.6084/m9.figshare.24679035.
